# *ZEB1* hypermethylation is associated with better prognosis in patients with colon cancer

**DOI:** 10.1186/s13148-023-01605-7

**Published:** 2023-12-13

**Authors:** Irene Fernandez-De-Los-Reyes, Marisa Gomez-Dorronsoro, Iñaki Monreal-Santesteban, Agustín Fernandez-Fernandez, Mario Fraga, Pablo Azcue, Laura Alonso, Beatriz Fernandez-Marlasca, Javier Suarez, Alicia Cordoba-Iturriagagoitia, David Guerrero-Setas

**Affiliations:** 1 Department of Pathology, Hospital Universitario de Navarra (HUN), Irunlarrea 3, 31008 Pamplona, Spain; 2 Molecular Pathology of Cancer Group, Navarrabiomed, Universidad Pública de Navarra (UPNA), Instituto de Investigación Sanitaria de Navarra (IdiSNA), Irunlarrea 3, 31008 Pamplona, Spain; 3 Oncogenetic and Hereditary Cancer Group, Universidad Pública de Navarra (UPNA), Instituto de Investigación Sanitaria de Navarra (IdiSNA), Irunlarrea 3, 31008 Pamplona, Spain; 4 Nanomaterials and Nanotechnology Research Center (CINN-CSIC), 33940 El Entrego, Spain; 5 Health Research Institute of Asturias (ISPA), 33011 Oviedo, Spain; 6 University Institute of Oncology (IUOPA), University of Oviedo, 33006 Oviedo, Spain; 7 Center for Biomedical Network Research on Rare Diseases (CIBERER), 28029 Madrid, Spain; 8 Department of Health Science, Public University of Navarra, Irunlarrea 3, 31008 Pamplona, Spain; 9 Department of Surgery, Hospital Universitario de Navarra (HUN), Irunlarrea 3, 31008 Pamplona, Spain

**Keywords:** CMS, Colon cancer, DNA methylation, Prognostic biomarker

## Abstract

**Background:**

Colon cancer (CC) is a heterogeneous disease that is categorized into four Consensus Molecular Subtypes (CMS) according to gene expression. Patients with loco-regional CC (stages II/III) lack prognostic factors, making it essential to analyze new molecular markers that can delineate more aggressive tumors. Aberrant methylation of genes that are essential in crucial mechanisms such as epithelial mesenchymal transition (EMT) contributes to tumor progression in CC. We evaluate the presence of hyper- and hypomethylation in subrogate IHC markers used for CMS classification (CDX2, FRMD6, HTR2B, ZEB1) of 144 stage II/III patients and CC cell lines by pyrosequencing. ZEB1 expression was also studied in control and shRNA-silenced CC cell lines and in paired normal tissue/tumors by quantitative PCR. The pattern of ZEB1 staining was also analyzed in methylated/unmethylated tumors by immunohistochemistry.

**Results:**

We describe for the first time the hypermethylation of *ZEB1* gene and the hypomethylation of the *FRMD6* gene in 32.6% and 50.9% of tumors, respectively. Additionally, we confirm the ZEB1 re-expression by epigenetic drugs in methylated cell lines. ZEB1 hypermethylation was more frequent in CMS1 patients and, more importantly, was a good prognostic factor related to disease-free survival (*p* = 0.015) and overall survival (*p* = 0.006) in our patient series, independently of other significant clinical parameters such as patient age, stage, lymph node involvement, and blood vessel and perineural invasion.

**Conclusions:**

Aberrant methylation is present in the subrogate genes used for CMS classification. Our results are the first evidence that *ZEB1* is hypermethylated in CC and that this alteration is an independent factor of good prognosis.

**Supplementary Information:**

The online version contains supplementary material available at 10.1186/s13148-023-01605-7.

## Introduction

Colon cancer (CC] is the third most prevalent type of cancer worldwide, with more than 1.1 million and 0.57 million new cases and cancer-related deaths, respectively, arising in 185 countries annually [1]. Among the wide range of risk factors, the biological features of CC and genetic and epigenetic tumor heterogeneity largely explain the different clinical outcomes. Advances in molecular pathology have allowed for the development of treatments such as the anti-EGFR monoclonal antibodies for metastatic CC patients (stage IV) without RAS mutations [2]. Nevertheless, the choice of chemotherapy treatment for patients with loco-regional CC (stages II–III) and bad prognostic factors is currently based only on histopathological and clinical factors [3]. For this reason, there is an urgent need to refine these prognostic factors in order to help clinicians stratify these patients more effectively.

Previously, CC has been categorized into four Consensus Molecular Subtypes (CMSs): CMS1 (MSI immune subtype), CMS2 (canonical subtype), CMS3 (metabolic subtype) and CMS4 (mesenchymal subtype) [4, 5]. This classification is based on the differential gene expression, detected by microarrays, of genes crucial to cancer onset and progression. However, logistic and economic constraints render the use of DNA microarrays for routine classification unfeasible for most Pathology Departments. Nevertheless, an advance in this field was achieved using a new approach based on a surrogate immunohistochemistry (IHC) panel that can be applied in routine clinical practice [6]. This panel comprises four IHC markers involved in crucial cell mechanisms: caudal-related homeobox 2 (CDX2), FERM domain-containing 6 (FRMD6), 5-hydroxytryptamine (serotonin) receptor 2B, G protein-coupled (HTR2B) and zinc finger E-box binding homeobox 1 (ZEB1).

There are few or no studies about the role of epigenetic alterations in the differential expression of subrogate *CDX2*, *FRMD6*, *HTR2B* and *ZEB1* genes and about their possible clinical value. Aberrant DNA methylation of promoter regions in genes is the best-known epigenetic modification. This alteration is involved in regulating the expression of a great variety of genes [7]. This mechanism can be altered in cancer and be of clinical utility in the early detection of a wide range of cancers, and in predicting their prognosis and response to treatment, for example, the response to temozolomide in glioma patients with hypermethylation of the *MGMT* DNA repair gene [8]. Nevertheless, no aberrantly methylated genes with prognostic value have been exploited in clinical practice to treat CC patients [9].

*CDX2* gene is known to be hypermethylated in colorectal cancer, but few attempts have been made to determine its clinical value in CC [10]. It encodes a homeobox transcription factor that plays an important role in the development and maintenance of the intestinal tract and is used as an IHC marker to distinguish between adenocarcinomas of colorectal origin and those arising in other organs. It inhibits Wnt signaling and consequently the epithelial–mesenchymal transition (EMT) associated with tumor initiation, invasion, metastasis, and resistance to therapy [11]; *CDX2* hypermethylation is frequent in late stages of lung cancer [12] and plays an important role in the activation of lung cancer cell proliferation by suppressing Wnt signaling [13].

The *FRMD6* gene is also altered in cancer but the causes of its aberrant expression have not been studied. FRMD6 protein can bind to actin filaments, thereby regulating actomyosin contractility in epithelial cell–cell junction complexes in order to maintain epithelial structure [14]. FRMD6 has been identified as an upstream regulator of the Hippo signaling cascade, which regulates cell contact inhibition, apoptosis and proliferation, which themselves are known to be deregulated in CC [15] and other cancers [16].

The HTR2B receptor binds its ligand serotonin activating the GNAQ, GNA11 and GNA13 proteins that participate in cell proliferation and survival through the activation of Janus kinase/signal transducer and activator of transcription (JAK/STAT) and RAF/mitogen-activated protein kinase (MEK)/ERK signal-transduction pathways, among other [17]. HTR2B gene has been described as an oncogene in uveal melanoma [18], among other solid tumors [19], and as a tumor suppressor gene in ovarian cancers [20].

Finally, ZEB1 belongs to the EMT-zing finger transcription factor family and is involved in crucial mechanisms related to the formation and development of the organs in the embryonic development, fibrosis and tumor progression [21]. It is crucial in promoting EMT in cancer, including CC [22]; its expression is inhibited by miR200, which is activated by suppressor gene *TP53* [23]; ZEB1 is known to be involved in regulating key factors in malignant cells at the invasive front of carcinomas, conferring a proinvasive and stem-like phenotype on cancer cells, as well as leading to a worse clinical prognosis in several human cancers [24].

ZEB1 is known to participate in important epigenetic mechanisms. Its overexpression causes the epigenetic deregulation of colon cancer cells via activation of chromatin mark H3K4me3 leading to EMT [25]. Additionally, dysregulation of ZEB1 antisense 1 (ZEB1-AS1), an outstanding cancer-related long non coding RNA (lncRNA) has been demonstrated to regulate ZEB1 expression and to play a pivotal role in tumorigenesis and progression [26]. Nevertheless, to our knowledge there are no studies about the possible regulation of the promoter of ZEB1 gene itself by aberrant methylation in CC.

The utility of CMS classification and the dearth of studies of epigenetic alterations in the subrogate genes prompted us to analyze the presence of aberrant methylation in those genes and examine its clinical value in stage II-III CC patients, who are characterized by the lack of prognostic biomarkers that are useful for their clinical follow-up.

## Results

### Study of aberrant methylation in subrogate genes

**Fig. 1:**
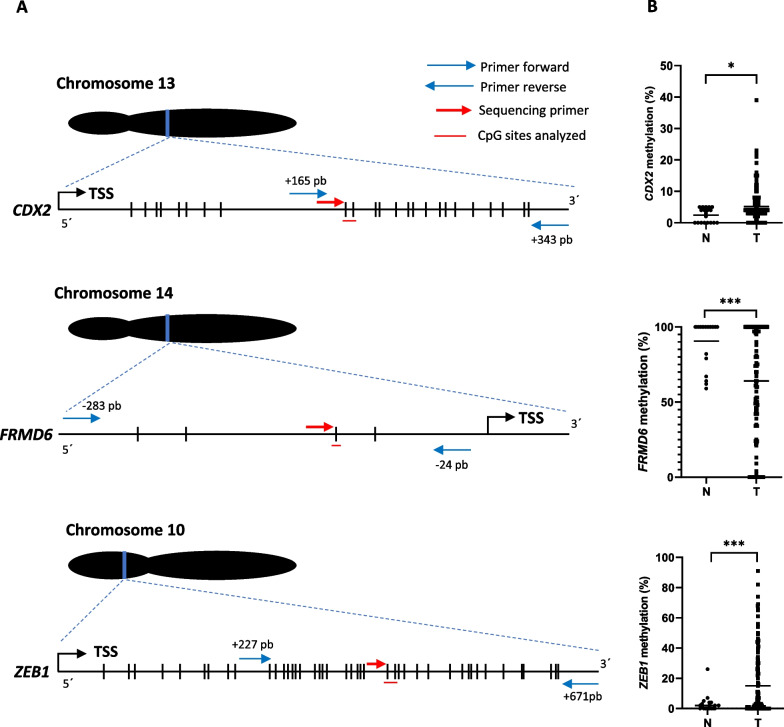
**A** Ideograms showing the location of the gene regions analyzed by pyrosequencing (PyroSeq) for *CDX2*, *FRMD6* and *ZEB1* genes, obtained from *UCSC Genome Browser (Human GRCh37/hg19)*. The transcription start sites (TSSs), and the location of the PCR and sequencing primers are displayed. CpGs sites are represented by vertical bars. **B** Average percentage of CpGs methylation values within *CDX2* and *ZEB1* genes, and CpG methylation value of *FRMD6* gene of each non-neoplastic colon (N) and each tumor (T) obtained by PyroSeq. The horizontal line represents the mean of both series (*p < 0.05, *** *p* < 0.001)

*CDX2*, *FRMD6* and *ZEB1* methylation could not be analyzed in 11.1%, 22.2% and 10.4% of the tumors, respectively, probably due to the effect of formalin fixation on the tissue [27]. The mean values of the average methylation levels of CpGs were 5.3% for CDX2, 59.5% for FRMD6 and 15.0% for ZEB1, respectively. The mean value was considered the threshold for distinguishing statistically between the unmethylated (less than the mean) and methylated (greater or equal to the mean) than status of each gene in normal and tumoral tissues. In the evaluable tumors, aberrant methylation was found for the three genes, with *CDX2* and *ZEB1* being hypermethylated in 32.8% and 32.6%, respectively, and *FRMD6* being hypomethylated in 50.9% of the patients. Aberrant *CDX2*, *FRMD6* and *ZEB1* hypermethylation or hypomethylation was more frequent in tumoral than in normal tissue (*p* = 0.04, *p* = 0.0004, *p* = 0.0024, respectively) (Fig. 1B).

**Fig. 2:**
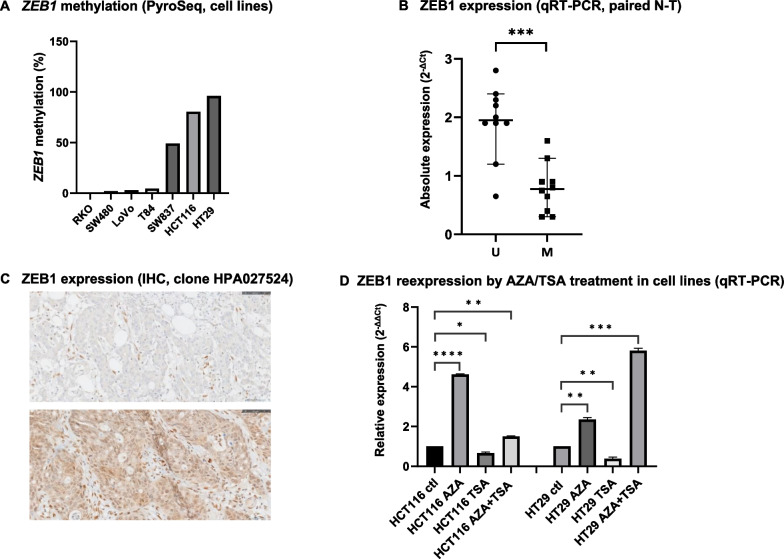
**A** Average percentage of CpGs methylation values within *ZEB1* gene in cell lines by increasing order, detected by pyrosequencing (PyroSeq). **B** ZEB1 expression in unmethylated (U) and highly methylated (M) paired normal tissues-tumors (N–T) (*n* = 10 for each group), calculated by 2^−ΔCt^ method. **C** Negative ZEB1 staining (methylated tumor), including positive nuclear staining in fibroblasts as internal control (up); positive nuclear ZEB1 staining in tumoral cells (unmethylated tumor) (down) (magnification: ×400).** D** Restoration of ZEB1 expression in HCT116 and HT29 cells by treatment of control cells (Ctl) with 5-aza-dC (AZA), trichostatin (TSA) and AZA + TSA, as calculated by 2^−ΔΔCt^ method (* *p* < 0.05, **p < 0.01, ***p < 0.001,****p < 0.0001)

HCT116, HT29 and SW837 cells were clearly methylated for *ZEB1* (80.5%, 96.0% and 49.0%, respectively). Conversely, *ZEB1* was completely unmethylated in RKO cells (0.0%) and scarcely methylated in LoVo, SW480 and T84 cells (3.0%, 2.0% and 4.5%, respectively) (Fig. 2A).

### Association between pathological and molecular parameters in CC

CC tumors with absent/low levels of CDX2 expression and a low percentage of positive tumor cells (< 25.0%) were associated with *CDX2* hypermethylation (*p* = 0.044 and *p* = 0.048, respectively) (Additional file 1: Fig. 1) and were preferentially of mesenchymal type and hMLH1/hPMS2 defective tumors (*p* < 0.005). Absent/low levels of expression are very frequent in stage III, less differentiated and right colon-sided CC tumors (*p* = 0.024, *p* = 0.006 and *p* = 0.093, respectively).

*FRMD6* hypomethylation was not associated with any of the variables included in the study, except for weaker PD-L1 expression (*p* = 0.012) analyzed previously by our group. It is remarkable that four of the five mesenchymal type tumors (80%) were hypomethylated for this gene compared with 49.5% of the epithelial ones (*p* = 0.182).

ZEB1 expression detected by qRT-PCR revealed that normalized ZEB1 expression of unmethylated tumors was higher than in methylated ones (*p* = 0.035) (Fig. 2B). ZEB1 expression detected by IHC in complete sections was well correlated with the findings obtained in TMAs in both groups of comparison (methylated *vs.* unmethylated tumors; high-grade *vs.* low-grade tumors) and produced no discordant results (*p* < 0.001). Lymphocytes and mesenchymal cells were used as internal positive controls of expression (Fig. 2C). There were no tumors with extensive positive ZEB1 expression, except isolated cell groups (< 5–10% of area of the slide) in more differentiated tumoral areas in contrast to no/lower levels of expression in undifferentiated areas. One of three tumors with a signet-ring phenotype characterized by its bad prognosis expressed a low level of ZEB1. *ZEB1* hypermethylation was associated with focal ZEB1 expression (*p* = 0.028). Finally, *ZEB1* hypermethylation was more frequent in the CMS1 subtype (*p* = 0.072), with a clear association of this epigenetic alteration with the pathological (null) expression of hMLH1 and hPMS2 proteins (*p* = 0.040 and *p* = 0.022, respectively).

### In vitro study

The highest levels of ZEB1 expression were detected mainly in AZA (*p* < 0.0001) and AZA + TSA (*p* = 0.003) groups of treated HCT116 and HT-29 cells, respectively (Fig. 2D).

The transfected RKO cells showed a significantly lower level of ZEB1 expression, mainly with shZEB1_1 (*p* = 0.031) in comparison with shZEB1_2 (*p* = 0.078) and shZEB1_3 (*p* = 0.016), with the greatest difference compared with the control (Additional file 2: Fig. 2). SW620 and T84 cells did not re-express ZEB1, probably because ZEB1 expression is regulated by a different mechanism. It is notable that it was not possible to select transfected cells with these shRNAs to perform functional assays (cell migration, cell invasion, response to treatment) because ZEB1 knockdown by shRNAs induced cell death in the first few hours after transfection.

### Survival analysis

The median follow-up for DFS and OS was 5.30 and 5.48 years, respectively. The univariate analyses confirmed that factors such as age, tumor size, stage, lymph node involvement, vessel invasion and perineural invasion were associated with worse prognosis as indicated by DFS (*p* < 0.001, *p* < 0.001, *p* = 0.040, *p* = 0.017, *p* = 0.005 and *p* = 0.034, respectively) and OS (*p* < 0.001, *p* < 0.001, *p* = 0.042, *p* = 0.030 and *p* = 0.220, respectively). It is of particular note that CMS subtypes had differential prognoses as previously reported [28], that of CMS4 being the worst (*p* = 0.014).

**Fig. 3:**
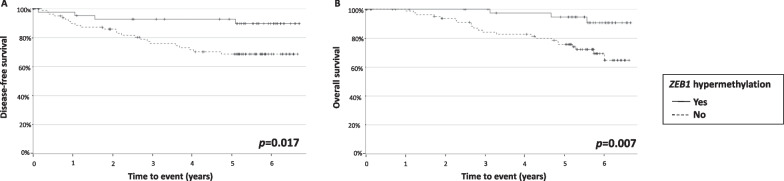
Univariate survival analysis. Kaplan–Meier curves stratified for (**A**) disease-free survival and (**B**) overall survival, stratified by *ZEB1* hypermethylation in all the patients. Log-rank *p* values are displayed

It is very striking that *ZEB1* hypermethylation was clearly associated with longer DFS and OS (*p* = 0.017 and *p* = 0.007, respectively) (Fig. 3). Therefore, the independent impact of *ZEB1* hypermethylation on DFS and OS, regardless of significant clinicopathological variables (patient age, stage, lymph node involvement, and blood vessel and perineural invasion), was tested in a Cox multivariate regression analysis. *ZEB1* hypermethylation was still significantly associated with longer DFS (*p* = 0.015) and OS (*p* = 0.006), irrespective of age, tumor size, stage, and blood vessel and perineural invasion (Table 1). The prognostic role of this alteration was maintained in the CMS2/3 subtypes (DFS: *p* = 0.023; OS: *p* = 0.009) (Additional file 3: Fig. 3).

**Table 1:** Multivariate Cox regression analysis of the risk of recurrence or death related to demographic and pathological variables in CC patients

		Disease-free survival		Overall survival	
Variable		HR (95% CI)	*p* value	HR (95% CI)	*p* value
Age		1.02 (0.98–1.06)	0.310	1.07 (1.02–1.12)	0.003
Tumor size		0.82 (0.62–1.08)	0.150	0.97 (0.79–1.19)	0.780
Stage	II	1	0.008	1	0.940
	III	36.33 (2.57–513)		0.005 (0.00–0.01)	
LNI	No	1	0.039	1	0.930
	Yes	0.07 (0.006–0.87)		472 (0.93–563)	
Blood vessel invasion	No	1	0.340	1	0.750
	Yes	1.67 (0.58–4.83)		1.51 (0.51–4.52)	
Perineural invasion	No	1	0.370	1	0.980
	Yes	1.56 (0.59–4.14)		0.99 (0.36–2.73)	
*ZEB1* hypermethylation	No	1	0.015	1	0.006
	Yes	0.22 (0.06–0.74)		0.18 (0.05–0.6)	

HR (95% CI): Hazard ratio, 95% confidence interval; LNI, Lymph node involvement

Conversely, *CDX2* hypermethylation and *FRMD6* hypomethylation were not of prognostic significance (Additional file 4: Fig. 4).

## Discussion

Colon cancer heterogeneity highlights the importance of undertaking studies to find new molecular markers. MMR proteins currently help distinguish between MSI tumors and MSS, with better prognosis and response to treatment for the first group [29]. The incorporation of the subrogate IHC panel (CDX2, FRMD6, HTR2B, ZEB1) to classify CC into CMS to detect mesenchymal type tumors characterized by their bad prognosis is an easy task [30]. Nevertheless, the subrogate panel is clearly incapable of distinguishing CMS2 from CMS3 patients. This is a problem, given that CMS2/CMS3 is the most numerous group (81.0% of our series). In this context, the discovery of key molecular alterations would allow new, clinically useful biomarkers to be proposed for the management of CC patients.

The detection of epigenetic alterations such as hypermethylation and hypomethylation of regulatory regions could explain the patterns of expression in tumors and may be clinically significant, as we have described previously in breast and cervical cancers, among other [31, 32]. It is worth noting that there have been no studies of epigenetic alterations of the *FRMD6* and *ZEB1* genes, which encode the proteins included in the panel, that are differentially expressed in colorectal cancer, as is demonstrated in *Human Protein Atlas* database [33]. Even less is known about the clinical role of these alterations in CC.

In the group of patients studied here, loss or absence of CDX2 expression was much more frequent in the CMS1 subtype but without prognostic significance [34], consistent with the findings of Baba et al. in sporadic CC [35]. The association between *CDX2* hypermethylation and lower levels of CDX2 expression is consistent with the first description of this epigenetic alteration in CC, which was detected by the less-informative methylation-specific PCR assay [36].

The clinical importance of the lack of CDX2 expression, measured as the level of mRNA or protein, has already been described in two reports [30, 37]. Less information is available about the clinical role of *CDX2* hypermethylation [38, 39]. In the group studied here, CDX2 expression was not correlated with clinicopathological variables except for a non-significant tendency for patients with methylated tumors (mainly in CMS2/3 patients) to display longer disease-free survival. This contrasts with the study by Jiang et al., which reported that *CDX2* hypermethylation was associated with a bad prognosis [39]. It is worth noting that all the stages were covered by the previous report; whereas, our present study examined only stage II/III tumors.

Very little is known about the *FRMD6* gene, expect that it is crucial to the Hippo pathway and therefore also to the EMT pathway. There is no agreement about what suppressor or oncogenic role *FRMD6* alteration might play in cancer. In keeping with its suppressor role, *FRMD6* mutations dysregulate the Hippo pathway by translocating the YAP/TAZ complex into the nucleus and thereby activating the expression of genes affecting key EMT genes (*ZEB1*, *Snail/Slug*, *Twist*) [40]. In line with these findings, low levels of FRMD6 expression are associated with worse prognosis in prostate cancer [41], and inhibition of the gene is directly related to progression of hepatocellular carcinoma [42]. Nevertheless, FRMD6 expression also contributes to cancer progression by activating the mTOR signaling pathway, similar to what occurs in lung cancer [43].

In the case of CC, FRMD6 is more strongly expressed in the serrated-type of colorectal cancer corresponding to colon cancer subtype 3 (CCS3) [44]. Unfortunately, in the group that we studied there were no cases available to examine with this morphology, probably due to the fact that serrated-type is more frequent in stage IV CC than in the stages II–III considered in this study [45]. FRMD6 was also reported to be upregulated in the poor survival CRC group by unknown causes [44, 46] and is also one of the panel of five key biomarkers of poor prognosis expressed in gastric cancer [47]. The mechanism underlying FRMD6 upregulation has not yet been determined. To our knowledge, our study is the first to report that *FRMD6* gene is highly methylated in normal colon tissue and hypomethylated in tumors. Overexpression could be mediated, at least in part, by DNA hypomethylation; in our study group, the lack of association between FRMD6 expression and hypomethylation could be related to components of post-transcriptional regulation of FRMD6 expression, such as phosphorylation events, or other epigenetic modifications (e.g., DNA methylation, histone acetylation, miRNA expression) [48].

In our study, neither *FRMD6* gene hypomethylation nor protein expression was associated with any clinicopathological variable, except for a clear association with a lower level of PD-L1 expression, a biomarker that predicts which patients with different types of cancer are more likely to respond to immunotherapy [49]. To our knowledge, this finding has not been reported elsewhere. Few studies have addressed the involvement of FMRD6 protein in the immune response; it is thought to be a neoantigen directly associated with the expression of HLA-A, and B and T cell activation characteristic of immune activated basal-like breast cancers with favorable prognosis [50].

*HTR2B* was recently described as being a suppressor gene whose mutations are related to the prognosis of squamous lung cancer [51] and metastasis in uveal melanoma [52]. Conversely, it has been described as an oncogene in CC whose aberrant activation promotes the TGF-beta pathway and metastasis [53]. There is no information about the presence of epigenetic alterations in this gene; it was not possible to study this here because the targeted CpG-rich region is very dense and the design of primers without CpGs in their sequence cannot be implemented. This is a frequent drawback in the analysis of FFPE samples, in which the starting material of study is so highly fragmented that the optimal amplicon length is restricted, thereby further limiting the options for primer placement [54].

ZEB1 is a crucial transcriptional activator of the transformation from epithelial phenotype to mesenchymal phenotype that promotes invasion, intravasation and dissemination to distant sites [55]. ZEB1 is upregulated in colorectal cancer, alongside other types of cancer such as those of the bladder, breast, stomach, pancreas and prostrate, and endometrial adenocarcinoma, oesophageal squamous cell carcinoma, head and neck squamous cell carcinoma, hepatocarcinoma, leiomyosarcoma and lung carcinoma [56, 57].The expression of this protein is also associated with resistance to oxaliplatin chemotherapy widely used in the clinical treatment of CC [58].

In the group studied here *ZEB1* hypermethylation, which was associated with a lower level of ZEB1 expression, was clearly associated with better prognosis, as indicated by DFS and OS, independently of other significant variables. This important finding also pertains to the CMS2/3 subgroup, which could enable clinicians to stratify this heterogeneous group of patients into groups with different degrees of risk of relapse or of death. These findings are consistent with those of Lindner et al. about the worse prognosis correlated with the high expression of ZEB1 [25]. The good prognostic role of this epigenetic alteration leading to ZEB1 silencing confirms the important role of this protein in the progression of CC.

Additionally, *ZEB1* hypermethylation is associated with the CMS1 subtype, characterized by its high degree of immune infiltration and better prognosis confirmed in our sample. It is notable that the only patient with the CMS1 subtype who died displayed an unmethylated promoter. The influence of ZEB1 expression in immune infiltration has been well studied, with reports on the inhibition of immune response exerted by this protein in melanoma and lung cancer [59, 60]. The role of *ZEB1* hypermethylation in this context and its influence on conventional or experimental treatments [61, 62] should therefore be investigated further.

## Conclusions

To our knowledge, this is the first report of aberrant methylation of the subrogate genes *FRMD6* and *ZEB1* being used for CMS classification. More importantly, we describe here for the first time the role of *ZEB1* hypermethylation as a crucial biomarker for the better prognosis of CC patients, as represented by disease-free survival and overall survival.

## Materials and methods

### Group of study

The group of patients studied consisted of 144 patients diagnosed with stage II (80 patients, 55.6%) and stage III (64 patients, 44.4%) sporadic CC between 2012 and 2013 in the Pathology Department of the Hospital Universitario de Navarra (Navarra Public Health System). All patients underwent surgical resection and tumors were staged according to their size, lymph node involvement and distant metastasis, following the most recent recommendations [63]. None of the patients had received radiation or chemotherapy before surgery. The study was approved by the Regional Clinical Research Ethics Committee (CEIC) (*Pyto2017/51 Cod. MOL_CRC,* 15 May 2018). The diagnosis of these tumors was confirmed following microscopic inspection by a certified pathologist with expertise and specialism in colon pathology (M.G.D.).

Tumors were classified by the subrogate IHC panel into CMS1 (MSI Immune), CMS2/CMS3 (Canonical/Metabolic) and CMS4 (Mesenchymal) in 18 (12.5%), 117 (81.3%) and nine patients (6.3%), respectively, based on previously established criteria [6].

Pathological and clinical characteristics are summarized in Table 2. Adjuvant chemotherapy was administered in 53 patients (37.9%), preferentially in stage III patients (81.1%) compared with stage II patients (18.9%), according to standard procedures. Follow-up included a physical and clinical examination every 4 months. During follow-up, 25 (17.4%) patients died of the disease and 26 (18.1%) died of other causes.

**Table 2:** Pathological and clinical characteristics of CC patient series

Variable		Frequency *n* (%)
*Age (years)*		
Mean		72.2
Range		48–93
*Gender*		
Female		46/144 (31.9)
Male		98/144 (68.1)
*Tumor location*		
Right colon		79/144 (54.9)
Left colon		65/144 (45.1)
*Histologic type*		
ADC NOS*		125/144 (86.8)
Colloid		18/144 (12.5)
SRCC**		1/144 (0.7)
*Differentiation grade*		
Well differentiated		118/144 (81.9)
Moderately-Poorly differentiated		26/144 (18.1)
*Tumor size (cm)*		
Mean		4.51
Range		1.5–13
*Lymph node involvement*		
No		81/144 (56.3)
Yes		63/144 (43.8)
*Stage*		
II		80/144 (55.6)
III		64/144 (44.4)
*Lymphatic vascular invasion*		
Negative		108/144 (75.0)
Positive		36/144 (25.0)
*Blood vessel invasion*		
Negative		102/144 (70.8)
Positive		42/144 (29.2)
*Perineural invasion*		
Negative		112/144 (77.8)
Positive		32/144 (22.2)
*Chemotherapy*		
No		53/144 (36.8)
Yes		67/144 (60.4)
Not valuable		4/144 (2.8)
*Recurrence*		
No		116/144 (80.6)
Yes		27/144 (18.8)
Not valuable		1/144 (0.7)
*Exitus*		
No		113/144 (78.5)
Yes		31/144 (21.5)

*ADC NOS: Adenocarcinoma not otherwise specified; **SRCC: Signet ring cell carcinoma

### Immunohistochemical study

Three-μm sections of tissue microarrays (TMAs) blocks harboring four tumor-carrying cores selected by the pathologist were placed on slides and then deparaffinized, hydrated and treated to block endogenous peroxidase activity using Vision Biosystems Bond-Max (Leica, Wetzlar, Germany) and Bench-Mark XT Ventana (Roche, Basel, Switzerland) automatic immunostaining apparatus, as previously published [64]. These slides were incubated with the appropriate primary antibodies against mismatch repair proteins-MMR (MLH1, MSH2, MSH6) and against proteins of the subrogate panel (CDX2, FRMD6, HTR2B and ZEB1) under the conditions summarized in Additional file 5: Table 1. In each TMA, normal colonic mucosa and stromal ovary tissue were included as the positive and negative control, respectively. A minimum of 500 tumor cells per tumor were counted by two independent expert pathologists. To evaluate the immunostaining pattern of the four proteins, we used the online test for CCR classification (https://crcclassifier.shinyapps.io/appTesting/). Expression of nuclear CDX2 and cytoplasmic FRMD6 was evaluated by categorizing counts of positive tumor cells into three categories (null/low number of positive cells: no expression or expression in fewer than 25% of cells; intermediate: expression in 26–55% of cells; high: expression in 56–100% of cells) and the intensity of the expression (low, intermediate and high). They were considered separate variables for the purpose of comparison. Diffuse CDX2 expression present in normal mucosa was used as a positive internal control and reference for the intensity of expression. Cytoplasmic HTR2B expression was evaluated in terms of its intensity, as in the case of CDX2/FRMD6. Nuclear ZEB1 was scored as its presence or absence. ZEB1 expression was also measured by IHC in complete sections of two groups of patients. The first group (16 tumors) included eight completely unmethylated (0.0%) tumors and eight highly methylated (> 50% methylation) tumors. The second group consisted of high-grade tumors (those with < 50% of glandular differentiation) (15 tumors). Programmed death ligand 1 (PD-L1), previously analyzed by our group [28], and P53 proteins were also evaluated in TMAs, following previous criteria [28, 65].

### DNA extraction from cell lines and tissue

DNA was extracted from 0.5 × 10^6^ cells in the case of cell lines, while for tumoral/normal tissues, it was obtained by QIAamp DNA Tissue kit (Qiagen, Hilden, Germany) from a representative area with more than 70% of tumoral cells in 5-μm-thick formalin-fixed, paraffin-embedded (FFPE) sections selected by the pathologist. DNA concentration was measured using an Invitrogen™ Qubit™ 3 Fluorometer (Thermo Fisher Scientific, Waltham, MA, USA).

### Pyrosequencing of subrogate genes

DNA methylation levels for *CDX2*, *FRMD6* and *ZEB1* genes were analyzed by bisulfite pyrosequencing in 144 patient tumor samples and in 40 paired normal tissues. The sets of primers for PCR amplification of analyzed CpGs (two positions in the case of *CDX2* and *ZEB1*, and one position in the case of *FRMD6*) and sequencing for each gene (Fig. 1A, 1B, 1C) were designed using the specific PyroMark assay design software (version 2.0.01.15; Qiagen, Hilden, Germany). We considered the same genomic regions of the *CDX2* and *ZEB1* genes that were previously analyzed by the methylation-specific PCR (MSP) method or Illumina methylation arrays, respectively [66, 67]. In the case of *FRMD6,* primers were designed to cover the promoter region. The location of the analyzed CpGs with respect to *GRCh37/hg19*, the primer sequences and the PCR conditions are included in Additional file 5: Table 2. It was not possible to design primers for HTR2B due to the high CpG density.

Bisulfite modification of DNA was performed with an EZ DNA methylation-gold kit (Zymo Research Irvine, CA, USA), following the manufacturer’s instructions. PCR amplification, pyrosequencing and methylation quantification were performed using PyroMark Q96 reagents in a PyroMark Q96 ID (Qiagen, Hilden, Germany). The average methylation percentage of CpGs of each gene was calculated for each tumor and normal tissue.

The survival of patients bearing these genes was analyzed (see below) to test the clinical value of the aberrant gene methylation of subrogate genes.

### In vitro* treatments*

Additional in vitro and molecular studies were carried out to test the biological value of ZEB1 hypermethylation. A panel of seven cell lines derived from colon cancer (HCT116, HT29, LoVo, RKO, SW480, SW837 and T84) was used to study ZEB1 (kindly donated by Dr. Arozarena, Navarrabiomed, Spain). All these cell lines were grown in DMEM, supplemented with 10% fetal bovine serum and 1.0% penicillin/streptomycin (all from Life Technologies, Carlsbad, CA, USA) at 37 °C in a humidified atmosphere with 5% CO_2_. The basal level of ZEB1 methylation was assessed in all cell lines.

Two highly methylated cell lines (HCT116, HT29) and one demethylated (RKO) cell line were treated at low passage with the demethylating agent 5-aza-2′-deoxycytidine (AZA) and the histone deacetylase inhibitor trichostatin A (TSA) (both from Sigma-Aldrich, St Louis, MO, USA). Briefly, cells were seeded at a density of 1 × 10^5^ cells/ml in six-well plates, allowed to attach overnight, and treated with 4 μM AZA for 72 h added freshly every 24 h, 300 nM TSA for 24 h, or the combination of the two drugs (4 μM AZA + 300 nM TSA) for the final 24 h, using PBS as a vehicle control.

### RNA extraction and quantitative reverse transcription PCR (qRT-PCR)

qRT-PCR was performed to check the restoration of ZEB1 expression in control and AZA + TSA-treated CC-derived cell lines. Three replicates were performed for each experimental condition. This analysis was also performed in 20 paired paraffin tumor–normal tissues (10 methylated and 10 unmethylated) to check the differential expression of this marker in tissue.

To this end, total RNA was extracted and purified using the RecoverAll kit (Thermo Fisher Scientific, Waltham, MA, USA) following the manufacturer’s instructions. Five hundred nanograms of total RNA were retrotranscribed using a PrimeScript™ RT Reagent Kit (TaKaRa, Otsu, Japan) at 37 °C for 15 min and 85 °C for 5 s. One microliter of the resulting cDNA was placed in a 96-well plate with 0.5 μl TaqMan probes (ZEB1: Hs.PT.58.39178574, IDT, Coralville, Iowa, USA) and 19 μl of mix were included in the Premix ExTaq™ kit (TaKaRa, Otsu, Japan). PCR amplification was performed in triplicate using the Quant Studio 12 K Flex (Life Technologies, Carlsbad, CA, USA) under thermal cycler conditions of 95 °C for 30 s and 40 cycles at 95 °C for 5 s and 60 °C for 34 s. Cycle threshold (Ct) values were calculated using Quant Studio software (Life Technologies, Carlsbad, CA, USA), using the reference housekeeping pseudogene-free ribosomal gene (18S rRNA: Hs.PT.39a.22214856.g, IDT Coralville, Iowa, USA), which shows little variation in basal expression in colon cancer [68, 69], as a normalization standard Absolute values of ZEB1 expression in normal tissues and tumoral tissues were calculated by ΔCt method (2^−ΔCt^) (Fig. 2B). The fold change in ZEB1 expression of each cell treatment (AZA, TSA, AZA + TSA) relative to the control value (ctl) was calculated by the ΔΔCt method (RQ = 2^−ΔΔCt^) (Fig. 2C).

### ZEB1 silencing in colon cancer cell lines

ZEB1 expression was silenced in cell lines positive for ZEB1 expression (RKO, SW620 and T84 cells) by short hairpin RNAs (shRNAs). For shRNA construction, three sequences targeting ZEB1 (shZEB1_1, shZEB1_2, shZEB1_3) and one scramble sequence were used (Additional file 5: Table 3). After inserting shRNAs into the pHIV1-SIREN-PuroR plasmid (kindly provided by Dr. Escors, Navarrabiomed), BamHI and EcoRI restriction enzymes (Life Technologies, Carlsbad, CA, USA) and T4 DNA ligase enzyme (New England Biolabs, Ipswich, MA, USA), respectively, were used to digest and ligate the construction. XL1-Blue Competent cells were then transformed with these three shRNA constructions. Plasmids were purified using the Qiagen Plasmid Midi kit (Qiagen, Hilden, Germany) and sequenced to check the ligation. Since the plasmid contained the puromycin-resistance gene for mammalian cell selection, cell sensitivity to this antibiotic (Thermo Fisher Scientific, Waltham, MA, USA) was tested for 5 days, and a concentration of 1 μg/ml was chosen as optimal from a range of possibilities. 5 × 10^4^ cells were seeded in six-well plates, allowed to attach overnight and then stably transfected with 1.2 µg of the plasmid of interest and 1:3 (v/v) FuGene HD (Promega, Madison, WI, USA) containing scramble, shZEB1_1, shZEB1_2 and shZEB1_3 in 60 µl of DMEM (Lonza Biologics, Basel, Switzerland), as previously described [70]. qRT-PCR was performed to check the silencing of ZEB1 expression with three replicates for each experimental condition (control, shZEB1_1, shZEB1_2, shZEB1_3).

### Statistical analysis

Associations between molecular (aberrant methylation, RNA expression), pathological and clinical variables of this retrospective study were assessed with the chi-square or Fisher’s exact test. Disease-free survival (DFS) and overall survival (OS) were analyzed in all CC patients. Survival curves were calculated using the Kaplan–Meier method and compared by univariate (log-rank) test. A multivariate Cox (proportional hazards) regression model was used to test the independent contribution of each variable to patient outcome. The proportional hazard ratio and 95% confidence interval (95% CI) were calculated for each factor. The hazard risk was adjusted for tumor stage and patient age. Statistical significance was concluded for values of *p* < 0.05 in all analyses.

### Supplementary Information

**Additional file 1: Fig. 1.** Negative IHC staining for a CDX2 methylated tumor (upper); positive tumor corresponding to an unmethylated CDX2 tumor (lower) in TMA sections (magnification: x400).

**Additional file 2: Fig. 2.** Inhibition of ZEB1 expression in RKO cells by shRNAs_ZEB1_1 and _3. Knockdown efficiency in control and silenced cells was also checked by qRT-PCR (absolute values: 2^−ΔCt^). (*p<0.05; **p<0.01; ***p<0.001).

**Additional file 3: Fig. 3.** Kaplan–Meier plots for disease-free survival (**A**) and overall survival (**B**) stratified by ZEB1 promoter hypermethylation status in the CMS2/3 group of patients.

**Additional file 4: Fig. 4.** Kaplan–Meier Curves for **A** disease-free survival (DFS) and** B** overall survival (OS) stratified by CDX2 hypermethylation status;** C** DFS and** D** OS stratified by FRMD6 hypomethylation status.

**Additional file 5: Table 1.** Antibodies used for the immunohistochemical analysis. **Table 2.** Primers and conditions used for bisulfite PCR and pyrosequencing. **Table 3.** Sequences of shRNAs used for ZEB1 silencing

## Data Availability

Not applicable.

## References

[1] Sung H, Ferlay J, Siegel RL, Laversanne M, Soerjomataram I, Jemal A, Bray F (2021). Global cancer statistics 2020: GLOBOCAN estimates of incidence and mortality worldwide for 36 cancers in 185 Countries. CA Cancer J Clin.

[2] Martinelli E, Ciardiello D, Martini G, Troiani T, Cardone C, Vitiello PP, Normanno N, Rachiglio AM, Maiello E, Latiano T (2020). Implementing anti-epidermal growth factor receptor (EGFR) therapy in metastatic colorectal cancer: challenges and future perspectives. Ann Oncol.

[3] NCCN Clinical Practice Guidelines in Oncology (NCCN Guidelines®) for Guideline Colon Cancer (Version 3.2022). © National Comprehensive Cancer Network, Inc. 2023. All rights reserved. Available from: https://www.nccn.org/professionals/physician_gls/pdf/colon.pdf

[4] Dienstmann R, Vermeulen L, Guinney J, Kopetz S, Tejpar S, Tabernero J (2017). Consensus molecular subtypes and the evolution of precision medicine in colorectal cancer. Nat Rev Cancer.

[5] Guinney J, Dienstmann R, Wang X, de Reynies A, Schlicker A, Soneson C, Marisa L, Roepman P, Nyamundanda G, Angelino P (2015). The consensus molecular subtypes of colorectal cancer. Nat Med.

[6] Trinh A, Trumpi K, De Sousa EMF, Wang X, de Jong JH, Fessler E, Kuppen PJK, Reimers MS, Swets M, Koopman M (2017). Practical and robust identification of molecular subtypes in colorectal cancer by immunohistochemistry. Clin Cancer Res.

[7] Martisova A, Holcakova J, Izadi N, Sebuyoya R, Hrstka R, Bartosik M (2021). DNA methylation in solid tumors: functions and methods of detection. Int J Mol Sci.

[8] Hegi ME, Diserens AC, Gorlia T, Hamou MF, de Tribolet N, Weller M, Kros JM, Hainfellner JA, Mason W, Mariani L (2005). MGMT gene silencing and benefit from temozolomide in glioblastoma. N Engl J Med.

[9] Essa HYS, Kusaf G, Yuruker O, Kalkan R (2022). Epigenetic alteration in colorectal cancer: a biomarker for diagnostic and therapeutic application. Glob Med Genet.

[10] Wang Y, Li Z, Li W, Liu S, Han B (2016). Methylation of promoter region of CDX2 gene in colorectal cancer. Oncol Lett.

[11] Kim JH, Kang GH (2014). Molecular and prognostic heterogeneity of microsatellite-unstable colorectal cancer. World J Gastroenterol.

[12] Selamat SA, Galler JS, Joshi AD, Fyfe MN, Campan M, Siegmund KD, Kerr KM, Laird-Offringa IA (2011). DNA methylation changes in atypical adenomatous hyperplasia, adenocarcinoma in situ, and lung adenocarcinoma. PLoS ONE.

[13] Liu X, Zhang X, Zhan Q, Brock MV, Herman JG, Guo M (2012). CDX2 serves as a Wnt signaling inhibitor and is frequently methylated in lung cancer. Cancer Biol Ther.

[14] Moleirinho S, Tilston-Lunel A, Angus L, Gunn-Moore F, Reynolds PA (2013). The expanding family of FERM proteins. Biochem J.

[15] Yin F, Dong J, Kang LI, Liu X (2021). Hippo-YAP signaling in digestive system tumors. Am J Cancer Res.

[16] Harvey KF, Zhang X, Thomas DM (2013). The Hippo pathway and human cancer. Nat Rev Cancer.

[17] Benhassine M, Le-Bel G, Guérin SL (2022). Contribution of the STAT family of transcription factors to the expression of the serotonin 2B (HTR2B) receptor in human uveal melanoma. Int J Mol Sci.

[18] Benhassine M, Guérin SL (2018). Transcription of the human 5-hydroxytryptamine receptor 2B (HTR2B) gene is under the regulatory influence of the transcription factors NFI and RUNX1 in human uveal melanoma. Int J Mol Sci.

[19] Peters MAM, Meijer C, Fehrmann RSN, Walenkamp AME, Kema IP, de Vries EGE, Harry Hollema H, Oosting SF (2020). Serotonin and dopamine receptor expression in solid tumours including rare cancers. Pathol Oncol Res.

[20] Henriksen R, Dizeyi N, Abrahamsson PA (2012). Expression of serotonin receptors 5-HT1A, 5-HT1B, 5-HT2B and 5-HT4 in ovary and in ovarian tumours. Anticancer Res.

[21] Cheng L, Zhou MY, Gu YJ, Chen L, Wang Y (2021). ZEB1: new advances in fibrosis and cancer. Mol Cell Biochem.

[22] Vu T, Datta PK (2017). Regulation of EMT in colorectal cancer: a culprit in metastasis. Cancers.

[23] Semenov O, Daks A, Fedorova O, Shuvalov O, Barlev NA (2022). Opposing roles of wild-type and mutant p53 in the process of epithelial to mesenchymal transition. Front Mol Biosci.

[24] Zhang Y, Xu L, Li A, Han X (2019). The roles of ZEB1 in tumorigenic progression and epigenetic modifications. Biomed Pharmacother.

[25] Lindner P, Paul S, Eckstein M, Hampel C, Muenzner JK, Erlenbach-Wuensch K, Ahmed HP, Mahadevan V, Brabletz T, Hartmann A (2020). EMT transcription factor ZEB1 alters the epigenetic landscape of colorectal cancer cells. Cell Death Dis.

[26] Li J, Li Z, Leng K, Xu Y, Ji D, Huang L, Cui Y, Jiang X (2018). ZEB1-AS1: a crucial cancer-related long non-coding RNA. Cell Prolif.

[27] Lu K, Ye W, Zhou L, Collins LB, Chen X, Gold A, Ball SM, Swenberg JA (2010). Structural characterization of formaldehyde-induced cross-links between amino acids and deoxynucleosides and their oligomers. J Am Chem Soc.

[28] Azcue P, Encío I, Guerrero Setas D, Suarez Alecha J, Galbete A, Mercado M, Vera R, Gomez-Dorronsoro ML (2021). PD-L1 as a prognostic factor in early-stage colon carcinoma within the immunohistochemical molecular subtype classification. Cancers.

[29] Taieb J, Svrcek M, Cohen R, Basile D, Tougeron D, Phelip JM (2022). Deficient mismatch repair/microsatellite unstable colorectal cancer: diagnosis, prognosis and treatment. Eur J Cancer.

[30] Pilati C, Taieb J, Balogoun R, Marisa L, de Reyniès A, Laurent-Puig P (2017). CDX2 prognostic value in stage II/III resected colon cancer is related to CMS classification. Ann Oncol.

[31] Perez-Janices N, Blanco-Luquin I, Torrea N, Liechtenstein T, Escors D, Cordoba A, Vicente-Garcia F, Jauregui I, De La Cruz S, Illarramendi JJ (2015). Differential involvement of RASSF2 hypermethylation in breast cancer subtypes and their prognosis. Oncotarget.

[32] Guerrero-Setas D, Perez-Janices N, Blanco-Fernandez L, Ojer A, Cambra K, Berdasco M, Esteller M, Maria-Ruiz S, Torrea N, Guarch R (2013). RASSF2 hypermethylation is present and related to shorter survival in squamous cervical cancer. Mod Pathol.

[33] Uhlén M, Fagerberg L, Hallström BM, Lindskog C, Oksvold P, Mardinoglu A, Sivertsson Å, Kampf C, Sjöstedt E, Asplund A (2015). Proteomics. Tissue-based map of the human proteome. Science.

[34] Konukiewitz B, Schmitt M, Silva M, Pohl J, Lang C, Steiger K, Halfter K, Engel J, Schlitter AM, Boxberg M (2021). Loss of CDX2 in colorectal cancer is associated with histopathologic subtypes and microsatellite instability but is prognostically inferior to hematoxylin-eosin-based morphologic parameters from the WHO classification. Br J Cancer.

[35] Baba Y, Nosho K, Shima K, Freed E, Irahara N, Philips J, Meyerhardt JA, Hornick JL, Shivdasani RA, Fuchs CS (2009). Relationship of CDX2 loss with molecular features and prognosis in colorectal cancer. Clin Cancer Res.

[36] Kawai H, Tomii K, Toyooka S, Yano M, Murakami M, Tsukuda K, Shimizu N (2005). Promoter methylation downregulates CDX2 expression in colorectal carcinomas. Oncol Rep.

[37] den Uil SH, de Wit M, Slebos RJC, Delis-van Diemen PM, Sanders J, Piersma SR, Pham TV, Coupé VMH, Bril H, Stockmann HBAC (2021). Quantitative analysis of CDX2 protein expression improves its clinical utility as a prognostic biomarker in stage II and III colon cancer. Eur J Cancer.

[38] Wang Y, Li Z, Li W, Liu S, Han B (2018). Methylation of CDX2 gene promoter in the prediction of treatment efficacy in colorectal cancer. Oncol Lett.

[39] Jiang G, Luo C, Sun M, Zhao Z, Li W, Chen K, Fan T (2016). Methylation of CDX2 as a predictor in poor clinical outcome of patients with colorectal cancer. Genet Test Mol Biomark.

[40] Akrida I, Bravou V, Papadaki H (2022). The deadly cross-talk between Hippo pathway and epithelial-mesenchymal transition (EMT) in cancer. Mol Biol Rep.

[41] Haldrup J, Strand SH, Cieza-Borrella C, Jakobsson ME, Riedel M, Norgaard M, Hedensted S, Dagnaes-Hansen F, Ulhoi BP, Eeles R (2021). FRMD6 has tumor suppressor functions in prostate cancer. Oncogene.

[42] Guan C, Chang Z, Gu X, Liu R (2019). MTA2 promotes HCC progression through repressing FRMD6, a key upstream component of hippo signaling pathway. Biochem Biophys Res Commun.

[43] Wang T, Guo H, Zhang L, Yu M, Li Q, Zhang J, Tang Y, Zhang H, Zhan J. FERM domain-containing protein FRMD6 activates the mTOR signaling pathway and promotes lung cancer progression. Front Med. 2023. 10.1007/s11684-022-0959-5 37060526

[44] De Sousa EMF, Wang X, Jansen M, Fessler E, Trinh A, de Rooij LP, de Jong JH, de Boer OJ, van Leersum R, Bijlsma MF (2013). Poor-prognosis colon cancer is defined by a molecularly distinct subtype and develops from serrated precursor lesions. Nat Med.

[45] García-Solano J, Pérez-Guillermo M, Conesa-Zamora P, Acosta-Ortega J, Trujillo-Santos J, Cerezuela-Fuentes P, Mäkinen MJ (2010). Clinicopathologic study of 85 colorectal serrated adenocarcinomas: further insights into the full recognition of a new subset of colorectal carcinoma. Hum Pathol.

[46] Abdul Aziz NA, Mokhtar NM, Harun R, Mollah MM, Mohamed Rose I, Sagap I, Tamil AM, Ngah WZW, Jamal R (2016). A 19-gene expression signature as a predictor of survival in colorectal cancer. BMC Med Genom.

[47] Liu D, Zhou B, Liu R (2020). A transcriptional co-expression network-based approach to identify prognostic biomarkers in gastric carcinoma. PeerJ.

[48] Goodall GJ, Wickramasinghe VO (2021). RNA in cancer. Nat Rev Cancer.

[49] Zhang H, Liu L, Liu J, Dang P, Hu S, Yuan W (2023). Roles of tumor-associated macrophages in anti-PD-1/PD-L1 immunotherapy for solid cancers. Mol Cancer.

[50] Noblejas-López MDM, Nieto-Jiménez C, Morcillo García S, Pérez-Peña J, Nuncia-Cantarero M, Andrés-Pretel F, Galán-Moya EM, Amir E, Pandiella A, Győrffy B (2019). Expression of MHC class I, HLA-A and HLA-B identifies immune-activated breast tumors with favorable outcome. Oncoimmunology.

[51] Bao L, Zhang Y, Wang J, Wang H, Dong N, Su X, Xu M, Wang X (2016). Variations of chromosome 2 gene expressions among patients with lung cancer or non-cancer. Cell Biol Toxicol.

[52] Zhang Y, Yang Y, Chen L, Zhang J (2014). Expression analysis of genes and pathways associated with liver metastases of the uveal melanoma. BMC Med Genet.

[53] Mao L, Xin F, Ren J, Xu S, Huang H, Zha X, Wen X, Gu G, Yang G, Cheng Y (2022). 5-HT2B-mediated serotonin activation in enterocytes suppresses colitis-associated cancer initiation and promotes cancer progression. Theranostics.

[54] Candiloro ILM, Mikeska T, Dobrovic A (2017). Assessing alternative base substitutions at primer CpG sites to optimise unbiased PCR amplification of methylated sequences. Clin Epigenet.

[55] De Craene B, Berx G (2013). Regulatory networks defining EMT during cancer initiation and progression. Nat Rev Cancer.

[56] Ruan L, Chen W, Zhao X, Fang N, Li T (2022). Predictive potentials of ZEB1-AS1 in colorectal cancer prognosis and their correlation with immunotherapy. J Oncol.

[57] Sánchez-Tilló E, Liu Y, de Barrios O, Siles L, Fanlo L, Cuatrecasas M, Darling DS, Dean DC, Castells A, Postigo A (2012). EMT-activating transcription factors in cancer: beyond EMT and tumor invasiveness. Cell Mol Life Sci.

[58] Guo C, Ma J, Deng G, Qu Y, Yin L, Li Y, Han Y, Cai C, Shen H, Zeng S (2017). ZEB1 promotes oxaliplatin resistance through the induction of epithelial—mesenchymal transition in colon cancer cells. J Cancer.

[59] Plaschka M, Benboubker V, Grimont M, Berthet J, Tonon L, Lopez J, Le-Bouar M, Balme B, Tondeur G, de la Fouchardière A (2022). ZEB1 transcription factor promotes immune escape in melanoma. J Immunother Cancer.

[60] Wang Z, Zhang L, Xu W, Li J, Liu Y, Zeng X, Zhong M, Zhu Y (2022). The multi-omics analysis of key genes regulating EGFR-TKI resistance, immune infiltration, SCLC transformation in EGFR-mutant NSCLC. J Inflamm Res.

[61] Farooqi AA, Fayyaz S, Poltronieri P, Calin G, Mallardo M (2022). Epigenetic deregulation in cancer: enzyme players and non-coding RNAs. Semin Cancer Biol.

[62] Lin MJ, Svensson-Arvelund J, Lubitz GS, Marabelle A, Melero I, Brown BD, Brody JD (2022). Cancer vaccines: the next immunotherapy frontier. Nat Cancer.

[63] WHO Classification of Tumours Editorial Board. International Agency for Research on Cancer WHO classification of tumours of the digestive system, 5th edn. Lyon: International Agency for Research on Cancer; 2019.

[64] Azcue P, Guerrero Setas D, Encío I, Ibáñez-Beroiz B, Mercado M, Vera R, Gómez-Dorronsoro ML (2021). A novel prognostic biomarker panel for early-stage colon carcinoma. Cancers.

[65] Kim KM, Ahn AR, Park HS, Jang KY, Moon WS, Kang MJ, Ha GW, Lee MR, Chung MJ (2022). Clinical significance of p53 protein expression and TP53 variation status in colorectal cancer. BMC Cancer.

[66] Guo M, House MG, Suzuki H, Ye Y, Brock MV, Lu F, Liu Z, Rustgi AK, Herman JG (2007). Epigenetic silencing of CDX2 is a feature of squamous esophageal cancer. Int J Cancer.

[67] Ha YJ, Kim CW, Roh SA, Cho DH, Park JL, Kim SY, Kim JH, Choi EK, Kim YS, Kim JC (2015). Epigenetic regulation of KLHL34 predictive of pathologic response to preoperative chemoradiation therapy in rectal cancer patients. Int J Radiat Oncol Biol Phys.

[68] Ahmed FE, Ahmed NC, Vos PW, Bonnerup C, Atkins JN, Casey M, Nuovo GJ, Naziri W, Wiley JE, Allison RR (2013). Diagnostic microRNA markers to screen for sporadic human colon cancer in stool: I. Proof of principle. Cancer Genom Proteom.

[69] Korenkova V, Slyskova J, Novosadova V, Pizzamiglio S, Langerova L, Bjorkman J, Vycital O, Liska V, Levy M, Veskrna K (2016). The focus on sample quality: Influence of colon tissue collection on reliability of qPCR data. Sci Rep.

[70] Mendaza S, Ulazia-Garmendia A, Monreal-Santesteban I, Córdoba A, Azúa YR, Aguiar B, Beloqui R, Armendáriz P, Arriola M, Martín-Sánchez E (2020). ADAM12 is a potential therapeutic target regulated by hypomethylation in triple-negative breast cancer. Int J Mol Sci.

