# Supplementary figures and images for: ZEB1 hypermethylation is associated with better prognosis in patients with colon cancer
Source: Clin Epigenetics. 2023 Dec 13;15:193. doi: 10.1186/s13148-023-01605-7 (PMC10720242; doi:10.1186/s13148-023-01605-7)

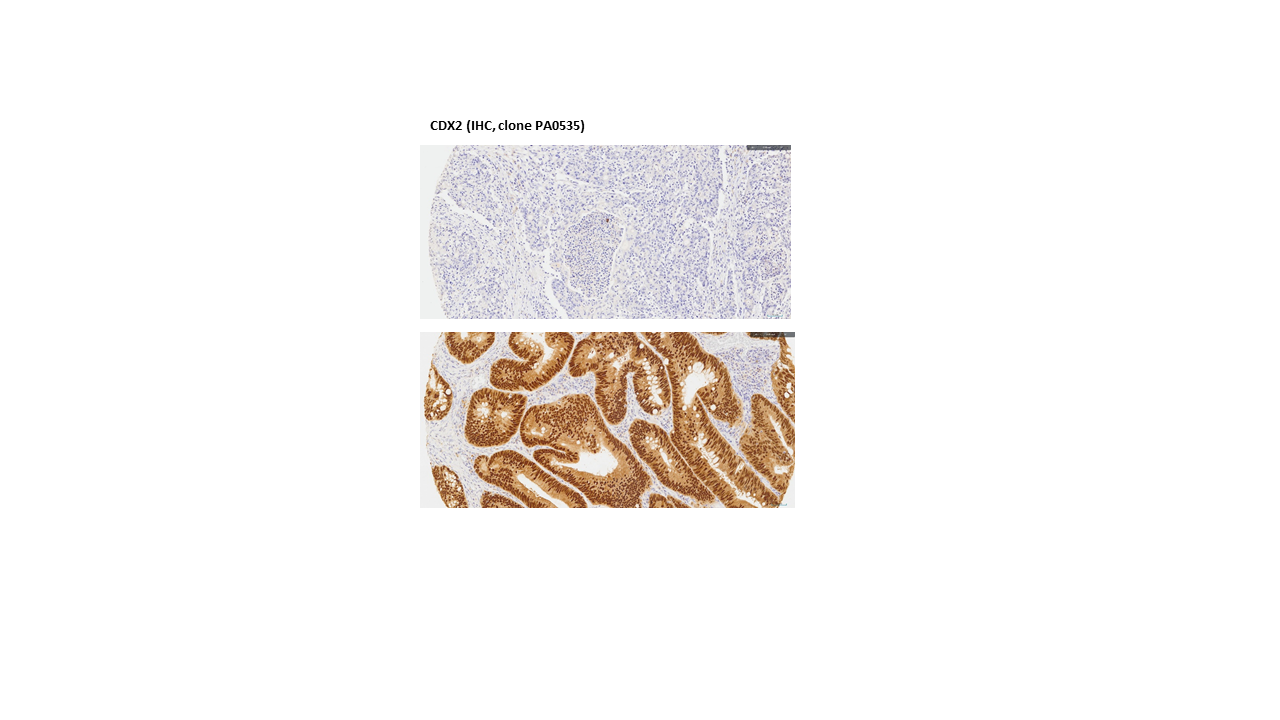

Supplement: Supplementary file 1 — Additional file 1: Fig. 1. Negative IHC staining for a CDX2 methylated tumor (upper); positive tumor corresponding to an unmethylated CDX2 tumor (lower) in TMA sections (magnification: x400). [file 13148_2023_1605_MOESM1_ESM.tif]

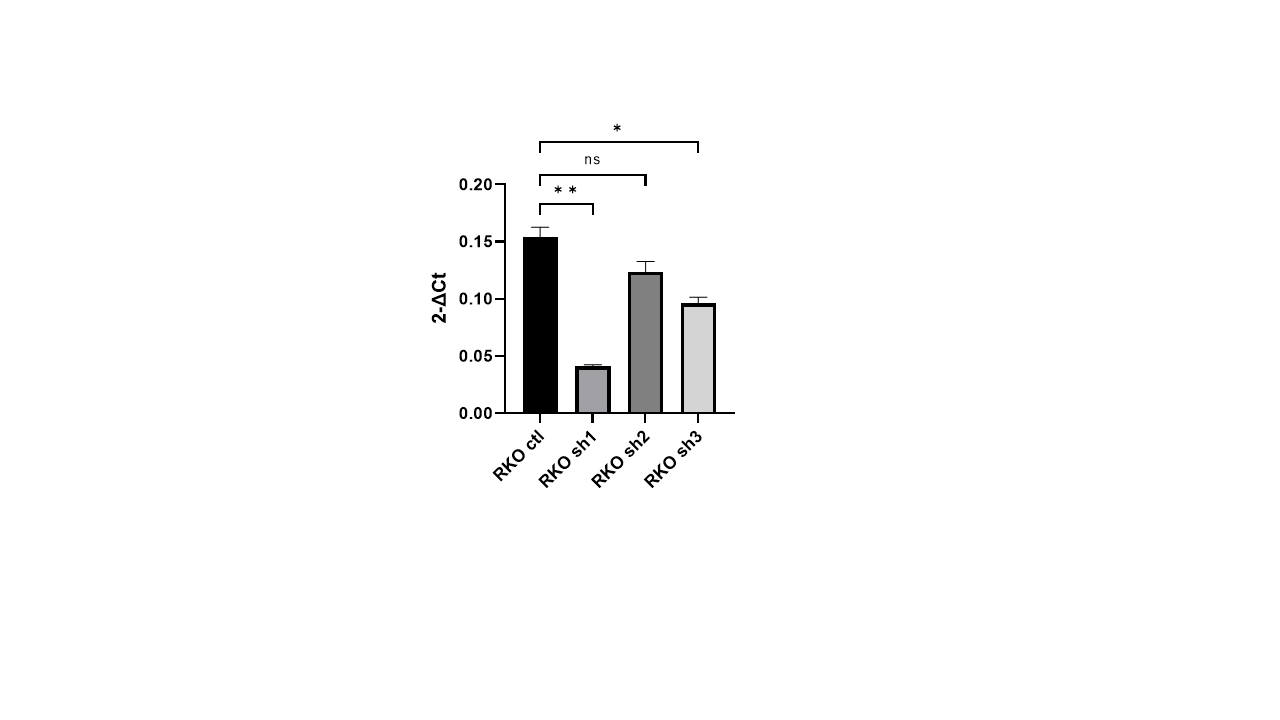

Supplement: Supplementary file 2 — Additional file 2: Fig. 2. Inhibition of ZEB1 expression in RKO cells by shRNAs_ZEB1_1 and _3. Knockdown efficiency in control and silenced cells was also checked by qRT-PCR (absolute values: 2−ΔCt). (*p<0.05; **p<0.01; ***p<0.001). [file 13148_2023_1605_MOESM2_ESM.tif]

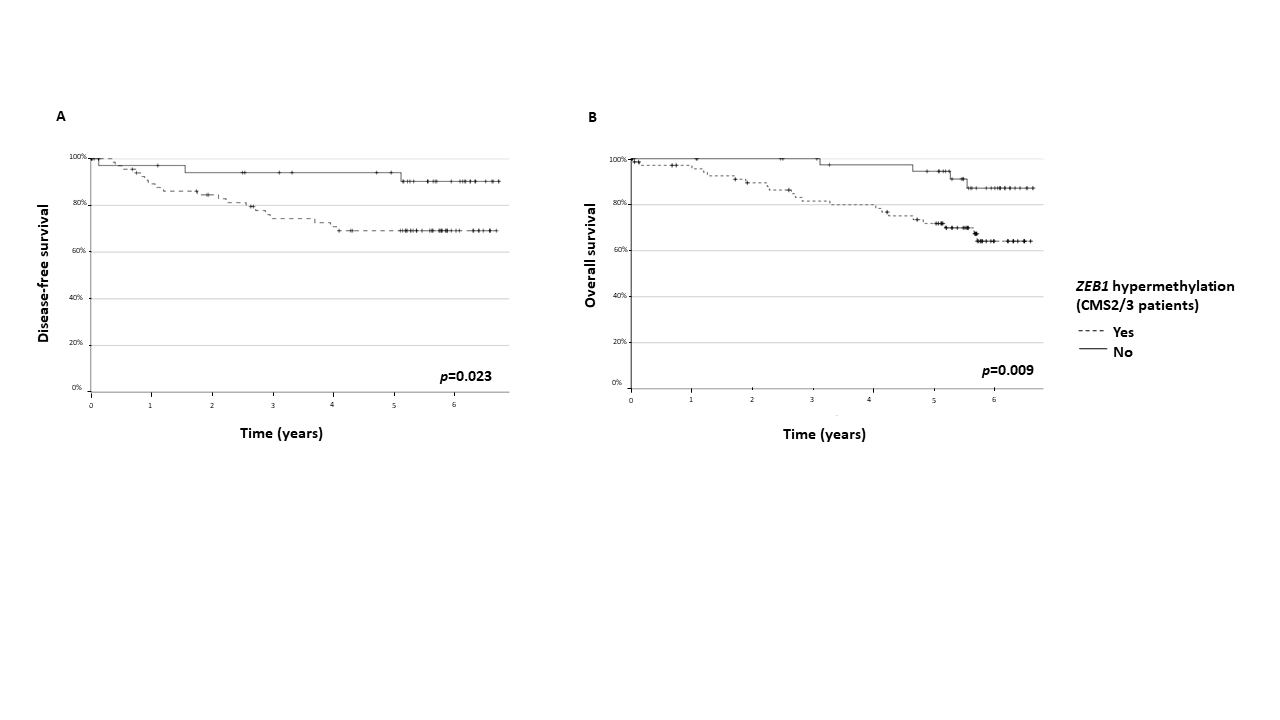

Supplement: Supplementary file 3 — Additional file 3: Fig. 3. Kaplan–Meier plots for disease-free survival (A) and overall survival (B) stratified by ZEB1 promoter hypermethylation status in the CMS2/3 group of patients. [file 13148_2023_1605_MOESM3_ESM.tif]

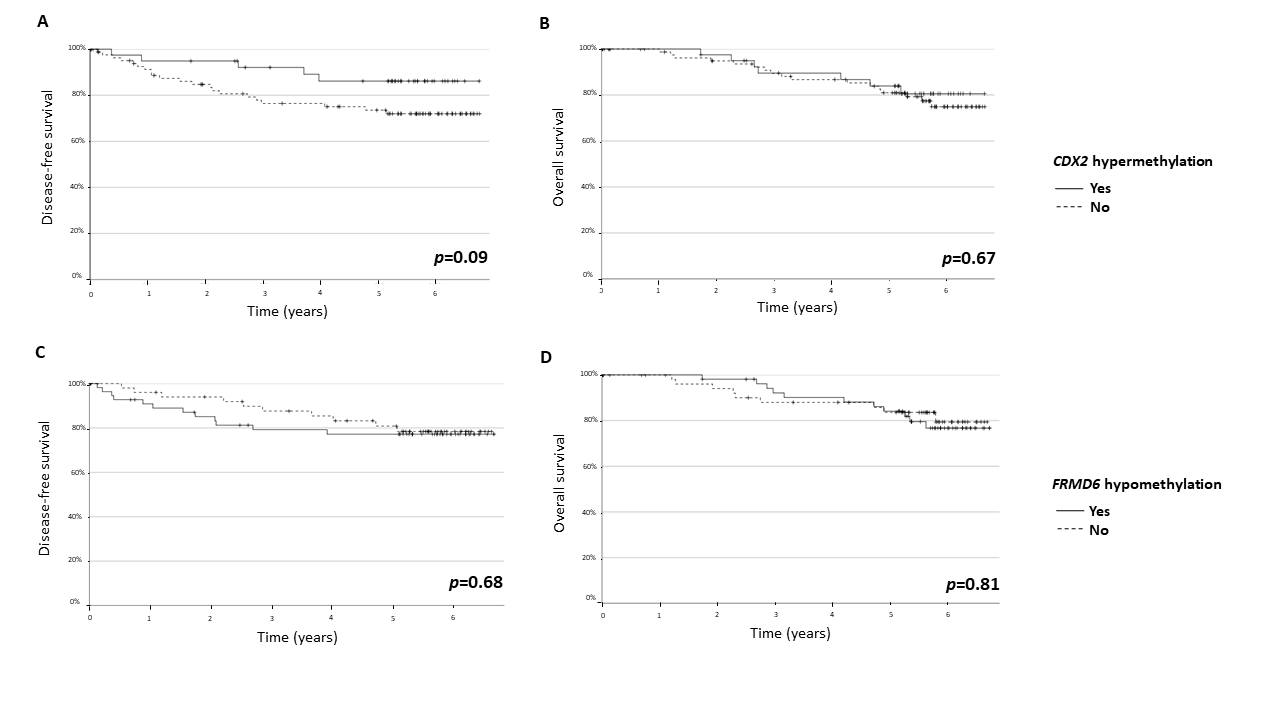

Supplement: Supplementary file 4 — Additional file 4: Fig. 4. Kaplan–Meier Curves for A disease-free survival (DFS) and B overall survival (OS) stratified by CDX2 hypermethylation status; C DFS and D OS stratified by FRMD6 hypomethylation status. [file 13148_2023_1605_MOESM4_ESM.tif]
