# Supplementary material for: ZEB1 hypermethylation is associated with better prognosis in patients with colon cancer
Source: Clin Epigenetics. 2023 Dec 13;15:193. doi: 10.1186/s13148-023-01605-7 (PMC10720242; doi:10.1186/s13148-023-01605-7)
Supplement: Supplementary file 5 — Additional file 5: Table 1. Antibodies used for the immunohistochemical analysis. Table 2. Primers and conditions used for bisulfite PCR and pyrosequencing. Table 3. Sequences of shRNAs used for ZEB1 silencing [file 13148_2023_1605_MOESM5_ESM.docx]

**Supplementary** **Table 1.** Antibodies used for the immunohistochemical analysis.

| **Antibody** | **Provider** | **Clone** | **Type** | **Dilution** | **Location** | **Immuno-stainer** |
| --- | --- | --- | --- | --- | --- | --- |
| MLH1 | Biocare | PA0610 | Monoclonal mouse | RTU | Nuclear | 1 |
| MSH2 | Calbiochem | FE-11 | Monoclonal mouse | 1:100 | Nuclear | 1 |
| MSH6 | Biocare | PM265AA | Monoclonal chimpanzee | RTU | Nuclear | 1 |
| PMS2 | Biocare | PM344AA | Monoclonal mouse | RTU | Nuclear | 1 |
| HTR2B | Sigma-Aldrich | HPA012867 | Polyclonal rabbit | 1:50 | Cytoplasmic | 2 |
| FRMD6 | Sigma-Aldrich | HPA001297 | Polyclonal rabbit | 1:50 | Cytoplasmic | 2 |
| ZEB1 | Sigma-Aldrich | HPA027524 | Polyclonal rabbit | 1:50 | Nuclear | 2 |
| CDX2 | Novocastra | PA0535 | Monoclonal mouse | RTU | Nuclear | 1 |
| PD-L1 | Roche | SP142 | Monoclonal mouse | RTU | Membrane | 2 |
| TP53 | Roche | DO-7 | Monoclonal mouse | RTU | Nuclear | 2 |

1: *Vision Leica Biosystems Bond-Max* automatic immunostainer; 2: *BenchMark XT Ventana* automatic inmunostainer (Roche); RTU: ready-to-use.

**Supplementary** **Table 2.** Primers and conditions used for bisulfite PCR and pyrosequencing.

| **Gene** | **CpGs location*** | **Forward (5'--3')** | **Reverse (5'--3')** | **Product size**  **(pairs of bases)** | **Annealing T** | **Sequencing (5'--3')** |
| --- | --- | --- | --- | --- | --- | --- |
| *CDX2* | CpG1- chr13:28,543,306  CpG2-chr13:28,543,311 | AGGGAGGAGGTAGGTTAGA | ACAAACCTCACCATACTACCTA | 179 | 60ºC | GTTAGAGGGAGGGAT |
| *FRMD6* | CpG-chr14:51,955,716 | TAGTGATTTTATGAATGAGGTGGATGTT | ATATAATTACTACATTACCCCTTCACA | 260 |  | ATAGTAATATTTTTGTAGATAGGT |
| *ZEB1* | CpG1-chr10:31,609,882  CpG2-chr10:31,609,891 | TGTTGATTGTTATTGTTTGGATAGTT | AAACAAAAAATCCCACACAAAATC | 445 |  | GTAGTTTAGGTTATATAAGGAAT |

*Location according to UCSC Genome Browser on Human (GRCh37/hg19)

**Supplementary** **Table 3.** Sequences of shRNAs used for ZEB1 silencing.

| **Gene** | **Forward (5' - 3')** |
| --- | --- |
| *sh_ZEB1_1_up* | GATCCGCCCCTCTCTGAAAGAACACATTACTCGAGTAATGTGTTCTTTCAGAGAGGGGTTTTTG |
| *sh_ZEB1_1_down* | AATTCAAAAACCCCTCTCTGAAAGAACACATTACTCGAGTAATGTGTTCTTTCAGAGAGGGGCG |
| *sh_ZEB1_2_up* | GATCCGCCGCTGTTGTTCTGCCAACAGTTCTCGAGAACTGTTGGCAGAACAACAGCGGTTTTTG |
| *sh_ZEB1_2_down* | AATTCAAAAACCGCTGTTGTTCTGCCAACAGTTCTCGAGAACTGTTGGCAGAACAACAGCGGCG |
| *sh_ZEB1_3_up* | GATCCGCCCGGCGCAATAACGTTACAAATCTCGAGATTTGTAACGTTATTGCGCCGGGTTTTTG |
| *sh_ZEB1_3_down* | AATTCAAAAACCCGGCGCAATAACGTTACAAATCTCGAGATTTGTAACGTTATTGCGCCGGGCG |
